# P-1733. Effectiveness of a Clinical Pharmacist-Led Electronic Medical Record-Based Antimicrobial Stewardship Intervention in Reducing the Overuse of Meropenem During Transition of Care: Evidence from a Tertiary Care Hospital in India

**DOI:** 10.1093/ofid/ofae631.1897

**Published:** 2025-01-29

**Authors:** Aravind R Nair

**Affiliations:** St. Joseph's College of Pharmacy, Pallickathodu, Kerala, India

## Abstract

**Background:**

In tertiary care hospitals, the overuse of broad-spectrum antibiotics like meropenem contributes to the rise of antimicrobial resistance, posing a significant public health concern, particularly in countries like India. Addressing this issue, this study evaluates the effectiveness of a clinical pharmacist-led electronic medical record-based antimicrobial stewardship intervention in curbing meropenem overuse during transitions of care at a tertiary care hospital in India.

**Methods:**

A prospective interventional study was conducted over six months at the tertiary care hospital. Trained clinical pharmacists led the intervention. Upon patients' transition from intensive care units (ICUs) to medical/surgical units, an active meropenem order triggered an alert in the EMR system. Pharmacists reviewed the order and liaised with the primary medical team for approval to continue meropenem therapy, documenting all communications and actions in the patient's EMR. Data on meropenem days of therapy (DOT) and related outcomes were collected pre- and post-intervention. Statistical analysis was performed to assess the impact of the intervention.

**Results:**

Out of 280 patients, 130 were in the pre-intervention group and 150 in the post-intervention group, with similar baseline characteristics. Pre-intervention, median meropenem DOT after transition was 4 days (IQR: 3-6), decreasing to 2 days post-intervention (IQR: 1-3) (p < 0.001), a 50% reduction. Median doses decreased from 8 (IQR: 6-10) to 5 (IQR: 3-7) post-intervention (p < 0.001). Total meropenem DOT significantly decreased post-intervention, from 600 to 375 days (p < 0.001). Furthermore, the acceptance rate of recommendations for de-escalation or discontinuation of meropenem therapy increased post-intervention, with 70% acceptance compared to 45% pre-intervention.

**Conclusion:**

In conclusion, the clinical pharmacist-led electronic medical record-based antimicrobial stewardship intervention effectively mitigated meropenem overuse during care transitions, highlighting its potential to optimize antibiotic utilization and combat antimicrobial resistance in Indian tertiary care settings.

**Disclosures:**

**All Authors**: No reported disclosures

